# Book Review: Martial Arts and Well-Being: Connecting Communities and Promoting Health

**DOI:** 10.3389/fsoc.2020.620880

**Published:** 2021-02-16

**Authors:** Danilo Contiero

**Affiliations:** University of the West of Scotland, Paisley, United Kingdom

**Keywords:** martial arts, health, book review, lifestyle and behavior, well-being

Danilo Contiero is a PhD candidate in clinical exercise, School of Health and Life Science, University of the West of Scotland. His Research Topics focus on healthy aging, physical activity, and martial arts. Danilo holds a BSc in Sport Science and an MSc in Sport Science for prevention and rehabilitation. Mr. Contiero is a member of the scientific and Dan committee for the “Ido Movement for Culture. Journal of Martial Arts Anthropology,” an active karate athlete, black belt 2 Dan, and karate instructor.

## Summary of The Book

The book “Martial Arts and Well-Being” by Fuller and Lloyd offers an interesting view about karate practitioners' health and well-being perception. It demonstrates how martial arts influence athletes' behavior and, in turn, their lifestyle and social activities. Results come from over 500 participants across all ages and ranges. The authors make very clear that the research does not aim to verify the health benefits *per se* but only the perception of them in martial arts athletes. Therefore, the study is based on different interviews and a qualitative methodology to access the participants' perceptions. The topics of well-being, martial arts, and social science seem really familiar to the authors, as Carol Fuller is a professor of education at the University of Reading, UK, and Viki Lloyd is the director of the Reading Acupuncture Clinic and lead instructor of a Karate and a Taijiquan school in Reading. The authors' experience, on the other hand, may have affected the sample's selection, as most of included participants were karate or Tai chi practitioners.

The book starts with an introduction about health-related problems of our century and how martial arts may help in the prevention and reduction of some of the most common illnesses. Different studies show the ability of martial arts to improve balance, cognitive functions, quality of life, and psychological health.

Very interesting is the link between martial arts and feeling of belonging to a group or community, as shown in the interviews at page 73 and supported also by other researches.

In chapter 2, the authors explore how behavior, lifestyle, and martial arts impact health and well-being, showing results from previous scientific researches, while in chapter 3, we find instructors of martial arts and their motivations to practice and learn. All other respondents (not teachers) involved in the study are included in chapter 4, which explores not only their motivations but also their perception of martial arts on health as the importance of the instructor in the learning process. Chapter 5 reports an analysis of interviews related to well-being. The authors make very clear in this section that they are not claiming a causal connection between well-being and martial arts in an empirical way, but they are trying to understand how athletes perceive this link. Chapter 6 draws the key themes found by the authors in the 511 interviews. Results underline the importance of martial arts for the participants' well-being perception and strongly support the socially constructed nature of health and well-being. In the last chapter, number 7, the authors show a summary of the significance of the research, with recommendations for policy and practice.

## Evaluation of The Book's Content

The book seems to be in line with the previous literature in the field of sport psychology, sport science, social science, and martial arts, and it offers a closer perspective to the participants' perception. Anyway, few limitations need to be addressed. The book title is about “Martial Arts,” but the sample of the study is based mostly on tai chi practitioners 51% and karate athletes 43%, followed by Qi Gong 21%, Kung Fu 6%, Aikido 4.5%, and Jujitsu 3% (Chapter 1, page 8). Therefore, this does not really help to have an overview on martial arts, but mostly on two of them, and mostly East Asian traditional martial arts. The second problem is related to the style of the art. In karate, for example, there are many styles, the most common are Shotokan, Wado-ryu, Shito-ryu, and Goju-ryu. As there is no protocol for karate training, every style, every club, and every instructor has a different way of teaching. One may argue that results may change according to the style and instructor. It is also very interesting to see that most of the interviews related to a link between martial arts and spiritualism or philosophical code are by tai chi students. This may lead to the question: Are philosophical and spiritual aspects proper for all martial arts? How much spiritualism is there in an MMA fight? Is that a martial art? Some may say yes and others say no, as at the moment, there is no universally agreed definition of “martial art.” Therefore, a definition and explanation of the meaning of martial arts by the author are necessary. At the same time, karate is a very popular sport, practiced by between 50 and 100 million people around the world, with a debut in the next Olympic Games. Anyway, of all the karate styles and associations, only the WKF karate has been approved by the Olympic committee. This is very important to underline as it makes very clear that also in the same martial art, styles, goals, and training change from school to school. Therefore, it is necessary to investigate if the philosophical, spiritual, and health perception reported by the participants have to be related to the martial art *per se* or more to a populistic and advertising popularization of martial arts, linked to spiritual myths of oriental culture.

## Discussion of The Book's Content

As an active athlete and instructor of the WKF, with more than 20 years of experience, the writer disagrees with any spiritual perception related to the martial art. This personal opinion that may look irrelevant in a scientific review can make sense if considered as a possible perception and view of a martial artist, which was not included in the study. Fuller and Lloyd may argue that it is not relevant if the participants' well-being perception is due to the martial art *per se* or to the populistic advertising of it, as the results will not change. On the other hand, this is very important to clarify if the research interest is to involve martial arts in the clinical field, as the authors aim.

Therefore, readers could definitely benefit from the book reading and gain more information about the importance of being active at any age. On the other hand, they are invited to look at the interview perception with a critical spirit.

Here are some of the questions that this book raised in the writer:

How much “divinization” has there been of martial arts and how much spiritualism was there in their origin, considering that some of them, like karate, were born from military arts?

Why don't we talk about this spiritualism in the western war/martial arts like the Greco–Roman wrestling? Art performed during the religious celebration of the God “Zeus,” therefore the link with the spiritual world should be intrinsic.

Why do most of the links with spiritualism in the interviews come from tai chi practitioners?

With these pungent and critical questions, I do not want to challenge the research results or the participants' perceptions that are definitely genuine and are needed for the love of martial arts and for their use and development in the clinical field. The reported health benefits of martial arts may be found in any other kind of exercise, leaving martial arts in a gray area without peculiar benefits when compared to other activities.

In conclusion, I recommend the book for those experts in the field of martial arts or social science and interested in understanding, through other people's experience, how our behavior and daily activities, not only martial arts, impact our perception of health. I would not recommend the book for those that are new to martial arts and may easily misunderstand the authors' message, overestimating or underestimating the value of the martial art they practice. I do not recommend the book to those who are looking for quantitative data and empirical definitive proofs about the benefits of martial arts on health. For research purposes, I think that the book brings a good value in social science and in psychology, for those researching the link between motivation and active behavior. On the other hand, I do not think that the book brings new findings in the field of martial arts as if in martial artists, the perception of health is affected by martial arts, in soccer players, it may be affected by soccer, and in runners, by the run, and so on, leaving martial arts at empty ends, with nothing more valuable than any other physical activity or sport. The research itself does not have a control group, and this makes the findings quite weak. I think that it is fair to consider the manuscript as an interesting book with a scientific approach, no more, no less.

## Author Contributions

The author confirms being the sole contributor of this work and has approved it for publication.

## Conflict of Interest

The author declares that the research was conducted in the absence of any commercial or financial relationships that could be construed as a potential conflict of interest.

